# Physicians’ Mutual Benefit Association of Texas

**Published:** 1885-09

**Authors:** 


Physicians' Mutual Benefit Association of Texas.—Dr. W. A. Morris, of Austin, has been elected President of this Association. Dr. Morris is, perhaps, the oldest active practitioner in Texas, having been steadily engaged in practice over half a century. This Association is simply an agreement between the members that, when one dies, all the others will contribute one dollar each to a fund for the deceased member's family, to be sent to the Secretary. No other expense except an annual fee of one dollar for stationery, postage, etc., and to pay the Secretary for his trouble. Send for blank applications to this office.

